# Distinct molecular adaptations underlie convergent phenotypic responses to mannose in normal and metastatic melanocytes

**DOI:** 10.1007/s11010-026-05713-9

**Published:** 2026-08-31

**Authors:** Nathália de Vasconcellos Racorti, Luis Roberto Fonseca Lima, Silvina Odete Bustos, Murilo Salardani, Uilla Barcick, Claudia Barbosa Ladeira de Campos, Bianca Alves Pauletti, Adriana Franco Paes Leme, Roger Chammas, André Zelanis

**Affiliations:** 1 Functional Proteomics Laboratory, Federal University of São Paulo – UNIFESP, São José dos Campos, São Paulo 12231-280 Brazil; 2 Grupo de Oncologia Experimental - Instituto do Câncer do Estado de São Paulo – ICESP, São Paulo, 01246-000 SP Brazil; 3 Laboratory of Biochemistry and Molecular and Cellular Biology of Fungi, Federal University of São Paulo – UNIFESP, São José dos Campos, São Paulo 12231-280 Brazil; 4 Laboratório Nacional de Biociências (LNBio), Centro Nacional de Pesquisa em Energia e Materiais (CNPEM), Campinas, São Paulo Brazil; 5 Faculdade de Medicina da Universidade de São Paulo, São Paulo, 01246-903 Brazil; 6 Functional Proteomics Laboratory, Department of Science and Technology, Federal University of São Paulo (ICT-UNIFESP), Rua Talim, 330, Room 202, São José dos Campos, São Paulo 12231-280 Brazil

**Keywords:** Melanoma, Phosphomannose isomerase, Phosphoproteomics, Mannose, Cancer metabolism

## Abstract

Mannose has emerged as a promising metabolic modulator capable of impairing tumor growth in phosphomannose isomerase (PMI)-deficient cancers. However, the molecular adaptations underlying the response of melanoma cells to mannose remain poorly understood. Here, we compared the phenotypic, proteomic, and phosphoproteomic responses of normal melanocytes (Melan-A) and metastatic melanoma cells (B16F10) cultured in glucose- or mannose-containing media. Both cell types exhibited similar phenotypic responses to mannose, including reduced growth, altered lactate production, and increased accumulation of acidic vesicles. Despite these shared phenotypes, proteomic and phosphoproteomic analyses revealed markedly distinct molecular adaptations. Mannose induced modest proteomic changes in normal melanocytes but extensive remodeling in metastatic melanoma cells, accompanied by profound phosphoregulatory remodeling. Substrate enrichment analysis based on curated signaling interactions identified phosphosubstrate signatures associated with predominantly homeostatic regulatory programs in normal melanocytes, whereas metastatic cells exhibited phosphosubstrate signatures associated with stress-responsive, survival, cell-cycle, and ubiquitin-mediated regulatory programs. Collectively, our findings demonstrate that similar phenotypic responses to mannose arise from fundamentally distinct molecular programs, highlighting cellular context as a key determinant of the adaptive response to mannose-induced metabolic stress in melanoma.

**Supplementary Information:**

The online version contains supplementary material available at https://doi.org/10.1007/s11010-026-05713-9.

## Introduction

Targeting metabolic pathways has become a central strategy in cancer therapy, because oncogenic reprogramming creates selective metabolic dependencies (for example, on glycolysis, glutaminolysis, or lipid synthesis) that can be exploited to selectively impair tumour growth and sensitize cancer cells to conventional treatments [1–4]. In this context, the hexose mannose, an epimer of glucose at C-2, has emerged as a metabolic modulator that impairs tumour growth by disrupting glycolytic metabolism in phospho mannose isomerase (PMI)-deficient cancers [5–8]. Mannose enters cells through the same facilitative glucose transporters (GLUTs) used by glucose and is rapidly phosphorylated by hexokinase to mannose-6-phosphate [9, 10]. Mannose-6-phosphate is then converted by PMI into fructose-6-phosphate, a glycolytic intermediate that can re-enter central carbon metabolism. The seminal work by González and colleagues [7] showed that mannose administration, when combined with conventional chemotherapy, alters the levels of anti-apoptotic Bcl-2 family proteins, thereby sensitizing cells to death; moreover, mannose susceptibility appears to depend on phosphomannose isomerase (PMI) expression levels. Accordingly, although PMI expression cannot be considered a prognostic biomarker, its expression and functional status may serve as predictive biomarkers of tumor sensitivity to mannose-based therapies [7, 8, 11].

Analysis of publicly available transcriptomic and proteomic datasets revealed a highly conserved pattern of phosphomannose isomerase (PMI, encoded by the *MPI* gene) expression across murine and human tissues [12, 13]. Among the tissues examined, skin consistently displays one of the lowest basal PMI expression levels, whereas the lung, pancreas, and testis exhibit comparatively high expression. Given the established relationship between PMI expression and cellular susceptibility to mannose, we reasoned that the intrinsically low expression of PMI in skin provides a suitable biological context in which to investigate the effects of mannose on melanocytic cells. Therefore, we employed murine models of normal melanocytes (Melan-A) [14] and metastatic melanoma (B16F10) [15] to investigate the biological and molecular responses to mannose exposure. Specifically, we sought to determine whether normal and malignant melanocytic cells, despite sharing a similarly low basal PMI expression profile, respond to mannose through common or distinct molecular adaptation programs.

## Methods

### Cell culture conditions and cell proliferation assays

Cell line B16F10 (CRL-6475™) [15], a mixture of spindle-shaped and epithelial-like cells from mouse skin, was obtained from the American Type Culture Collection (ATCC, USA). The cell line Melan-A is an immortalized line of pigmented melanocytes that was derived from normal epidermal melanoblasts obtained from embryos of inbred C57BL mice, as previously described [14]. Cells were cultured in Dulbecco’s Modified Eagle Medium (DMEM) (pH 7.0; Thermo Fisher, USA) supplemented with 2.0 g/L sodium bicarbonate, 100 mg/L streptomycin, 25 mg/L ampicillin, 2 mM glutamine, 10% fetal bovine serum (FBS; Vitrocell, Campinas, SP, Brazil), and 200 nM 12-O-tetradecanoyl phorbol-13-acetate (PMA; Sigma, USA). Although PMA is required for the growth of Melan-A cells, B16F10 cell proliferation is neither dependent on nor adversely affected by this tumor promoter. To ensure comparable culture conditions, 200 nM PMA was included in the culture medium for both Melan-A and B16F10 cells. The only excenption was in phosphoproteomics experiments, when PMA was omitted in the culture of B16F10 cells. Cultures were maintained at 37 °C in a humidified atmosphere containing 5% CO₂ and 95% air. The experimental conditions used in this study comprised two culture media compositions, namely: (1) DMEM with glucose (25 mM, final concentration) and (2) DMEM with mannose (25 mM, final concentration). Cells were grown in a humidified incubator at 37 °C, with 5% CO_2_. The evaluation of cell growth was performed in multiwell plates (6 wells,10 mm diameter, Sarstedt, Germany), as three independent experiments with cell lines supplemented with the different hexoses - Glucose (Glc) or Mannose (Man) (25 mM; final concentration). Cells were counted in quadruplicate using a automatic cell counter (Countess™ 3, ThermoFisher, USA). Cell lysates were obtained as described previously [16]. For the shotgun proteomics experiments, at least two biological replicates were used fo each experimental condition, as described in the Supplementary figure S1. Briefly, mechanical lysis was performed with a cell scrapper and 1 mL of cell lysis buffer (2% CHAPS, 150 mM NaCl in HEPES 50mM, pH 7.5) with the addition of a protease inhibitor cocktail (SIGMA*FAST*™, Sigma, USA). Lysates were incubated in an ice bath for 30 min under mild agitation. After centrifugation (14,000 *g*, 10 min, 4 °C), supernatants were removed, subjected to protein quantitation using the Bradford method [17] and stored at −80 °C until use. Cell lysates were independently obtained from three biological replicates derived from each cell line/experimental condition.

### Mannose-6-phosphate isomerase activity

Mannose-6-phosphate isomerase activity was determined using a colorimetric assay based on the conversion of mannose-6-phosphate to fructose-6-phosphate, as described previously [8]. Briefly, 50 µg of protein from cell lysates was diluted in reaction buffer (20 mM Tris, 0.5 mM ZnCl₂, pH 7.5), followed by the addition of mannose-6-phosphate (final concentration 222 µM). The reaction mixture was incubated for 10 min at 37 °C. Subsequently, cysteine-HCl was added (1.5% stock, 0.25% final concentration), followed by the addition of sulfuric acid (to a 60% final concentration) and an ethanolic carbazole solution (0.003% final concentration). The mixture was then incubated for 1 h at room temperature, and absorbance was measured at 560 nm. Assays were performed using cell lysates from three independent experiments.

### Quantitation of lactate in the culture media

Lactate levels were quantified using an enzymatic assay, following the manufacturer’s instructions (Labtest, Minas Gerais, Brazil). Conditioned media from three biological replicates of each cell line under all experimental conditions were analyzed. Differences in lactate concentration between glucose- and mannose-treated cells were assessed using an unpaired two-tailed Student’s *t*-test.

### Determination of acidic vesicular organelles (AVOs) by flow cytometry

Acidic vesicular organelles (AVOs) were evaluated using the acidotropic dye acridine orange, as previously described [18]. Cells were seeded at a density of 3 × 10⁴ cells per well in 12-well plates and maintained in DMEM supplemented with 10% FBS and 25 mM glucose. After 24 h, the medium was replaced with the respective experimental conditions: DMEM containing 25 mM glucose or 25 mM mannose. For three consecutive days, at 24-hour intervals, three wells per group (biological replicates) were collected for AVO staining and flow cytometric analysis. Briefly, cells were treated with 30 mM chloroquine for 1 h, followed by incubation with acridine orange (3 µg/mL; Thermo Fisher, USA) for 10 min at 37 °C. Cells were then washed with PBS and detached using 0.25% trypsin-EDTA. The cell suspension was centrifuged at 2,000 rpm for 2 min, and the pellet was resuspended in 200 µL of PBS. Samples were analyzed using an Attune NxT Flow Cytometer (Thermo Fisher, USA). As a positive control, cells were cultured in HBSS (an amino acid-free saline solution that induces starvation and promotes AVO formation). Differences between glucose- and mannose-treated cells were assessed using an unpaired two-tailed Student’s *t*-test.

### ***In-solution*** trypsin digestion

The *in-solution* trypsin digestion was performed according to the protocol described by Pessotti et al. [16]. Briefly, a solution of 6 M guanidine hydrochloride (GuHCl) was added to a sample of 100 *µ*g of protein from each cell lysate sample to a final concentration of 3 M GuHCl, followed by the addition of dithiothreitol (DTT) to a final concentration of 5 mM. The mixture was incubated at 65 °C for 60 min. Iodoacetamide (IAA) was then added to a final concentration of 15 mM, and the samples were incubated in the dark for 60 min at room temperature. DTT was added to a final concentration of 15 mM to quench the excess of IAA. The samples were cleaned up by adding ice-cold acetone (8 volumes) and methanol (1 volume), followed by the incubation of samples for 3 h at −80 °C. After centrifugation at 14,000 *g* for 10 min, protein pellets were washed twice with one volume of ice-cold methanol and then resolubilized with NaOH solution (final concentration of 2.5 mM), followed by the addition of 50 mM HEPES buffer, pH 7.5, to a final volume of 100 µL. Trypsin (Proteomics grade; Sigma, USA) was added at a 1:100 ratio (enzyme/substrate), and protein samples were incubated at 37 °C for 18 h. After desalting using C-18 cartridges (3 M Empore™ SPE Extraction disks, USA), peptide samples were dried in a SpeedVac and stored at −20 °C until nanoflow liquid chromatography/tandem mass spectrometry (LC − MS/MS) analysis.

### Phosphoprotein enrichment

Subconfluent cell cultures were exposed to the indicated experimental conditions (25 mM glucose or 25 mM mannose) for 24 h and subsequently lysed in cell lysis buffer (described above) supplemented with an EDTA-free protease inhibitor cocktail (Sigma, USA) and a phosphatase inhibitor (1 mM sodium orthovanadate, final concentration). At least two biological replicates were used fo each experimental condition, as described in the Supplementary figure S2. Phosphoproteins were enriched using the Pierce™ Phosphoprotein Enrichment Kit (Thermo Fisher Scientific, USA) according to the manufacturer’s instructions. The enriched protein fraction was concentrated using Vivaspin centrifugal filter units (Cytiva, USA; 3 kDa molecular weight cut-off), and protein concentration was determined by the Bradford assay. Samples were then subjected to in-solution tryptic digestion as described above.

### Liquid chromatography coupled to tandem mass spectrometry (LC-MS/MS)

Peptide concentrations were estimated colorimetricaly using the Pierce™ Quantitative Peptide Assays (Thermo Scientific, USA) and 1 microgram of peptides was injected for each biological sample and 2 microgram for peptides from phosphoenrichment experiment. Data-independent acquisition (DIA) analyses were performed using an Orbitrap Exploris 240 mass spectrometer (Thermo Fisher Scientific, Bremen, Germany) connected to the EASY-nLC system 1200 (Proxeon Biosystem, USA) through a Proxeon nanoelectrospray ion source. Peptides were separated by a 2–30% acetonitrile gradient during 75 min, using 80% acetonitrile in 0.1% formic acid, in a trap Acclaim PepMap 100 nanoViper 2PK C18 (2 cm × ID75 µm, 3 μm particle size, Thermo Scientific) in line with an analytical PepMap RSLC C18 ES 902 column (50 cm x id 75 μm, 2 μm particle size) at a flow rate of 250 nL/min. The nanoelectrospray voltage was set to 1.7 kV and the source temperature was 275 °C. The MS1 scan was acquired with a 120 K resolution within 375–1595 of m/z range, and the automatic gain control (AGC) value was set to 300% with automatic injection time. The MS2 scans were acquired with a 15 K resolution after fragmenting the precursor ions, which are obtained from 36 isolation windows, each spanning 16 m/z units from 400 to 975 m/z, an AGC target of 800% with the normalized collision energy (NCE) 27% of higher-energy collisional dissociation (HCD).

### Proteomics data processing

Raw DIA mass spectrometry data were processed in Spectronaut v18.6 (Biognosys) [19] using the directDIA+ (Deep) and BGS factory settings. Spectra were searched against the UniProt reference proteome database (UP000000589, *Mus musculus*, 54,822 protein entries; release February 2024).

Calibration was set to non-linear iRT calibration with precision iRT enabled. Identification was performed using 1% q-value cutoff on precursor and protein level whereas the maximum number of decoys was set to a fraction of 0.1 of library size. The mass tolerance for matching precursor and fragment ions was set to dynamic (default), which lets SN determine the optimal value. The quantification interference correction was enabled with at least three fragment ions used per peptide, major and minor group quantities were set to mean peptide and mean precursor quantity, respectively with Top3 group selection each. Label-free quantification of proteins was performed at MS2 level using cross-run normalization and no imputation strategy. For PTM search, probability cutoff was set to 0.75. Maximum 2 Trypsin/P missed cleavage and a minimum peptide length of 7 to 52 amino acids, carbamidomethylation was set as fixed modification and N-terminal acetylation, oxidation of methionine and phosphorylation were used as variable modifications [20].

### Bioinformatic analyses

Correlations between biological replicates were calculated in R [21] using the Pearson correlation coefficient. Proteomic differential abundance analysis was performed using the limma package after imputing missing quantitative values using the MinProb algorithm implemented in the *imputeLCMD* package (*q* = 0.01). Protein intensities were log_2_-transformed and fitted to a linear model followed by empirical Bayes moderation (*eBayes*). Proteins with an adjusted *P* value ≤ 0.05 and an absolute log_2_ fold change (|log_2_FC|) > 1 were considered differentially abundant. Volcano plots were generated using the EnhancedVolcano package. Phosphoproteomics data were further analyzed in R using custom scripts based on the tidyverse ecosystem. Quantitative intensity values from glucose (Glc) and mannose (Man) conditions were log_2_-transformed after replacing zero values with missing data (NA). Mean abundance was calculated for each condition, and differential phosphorylation was estimated as log_2_ fold change (Man vs. Glc). Sequence motif analysis was performed using ± 7 amino acid flanking regions surrounding regulated phosphosites with the ggseqlogo package. Positional residue enrichment was subsequently used to infer kinase classes based on canonical phosphorylation motifs [22]. Sites were classified as proline-directed when proline frequency at the + 1 position exceeded 30% of regulated sequences (MAPK/CDK-like motifs), as basophilic when the combined frequency of arginine or lysine at the − 3 position exceeded 40% (AKT/PKA-like motifs), and as acidophilic when the combined frequency of glutamate or aspartate at the + 3 position exceeded 30% (CK2-like motifs). An exploratory substrate enrichment analysis was performed to infer upstream signaling regulators associated with the observed phosphoproteomic changes. Mouse UniProt identifiers were converted to gene symbols using the *org.Mm.eg.db* package, and directed regulatory interactions were retrieved from *OmniPathR*. Because of the limited number of biological replicates, statistical testing was not performed at the individual phosphosite level. Instead, phosphoproteomic data were analyzed using a substrate enrichment strategy based on quantitative phosphosite measurements. Log_2_ fold changes were calculated for quantified phosphosites and subsequently integrated with curated regulator–substrate interactions retrieved from OmniPath. Only directed stimulatory or inhibitory interactions were retained to build the regulator–substrate interaction network. For each upstream regulator represented by at least three quantified downstream substrates, a standardized *z*-score was calculated from the mean substrate log_2_ fold change relative to the global phosphosite distribution. Positive *z*-scores indicated preferential enrichment under mannose conditions, whereas negative *z*-scores indicated preferential enrichment under glucose conditions. The highest-ranking upstream regulators were visualized as bar plots and regulator–substrate interaction networks generated using *igraph* and *ggraph*, with edge width proportional to the absolute substrate log_2_ fold change and node connectivity representing interaction degree.

## Results and discussion

### Normal and transformed mouse melanocytes exhibit similar sensitivity to mannose

**Fig. 1:**
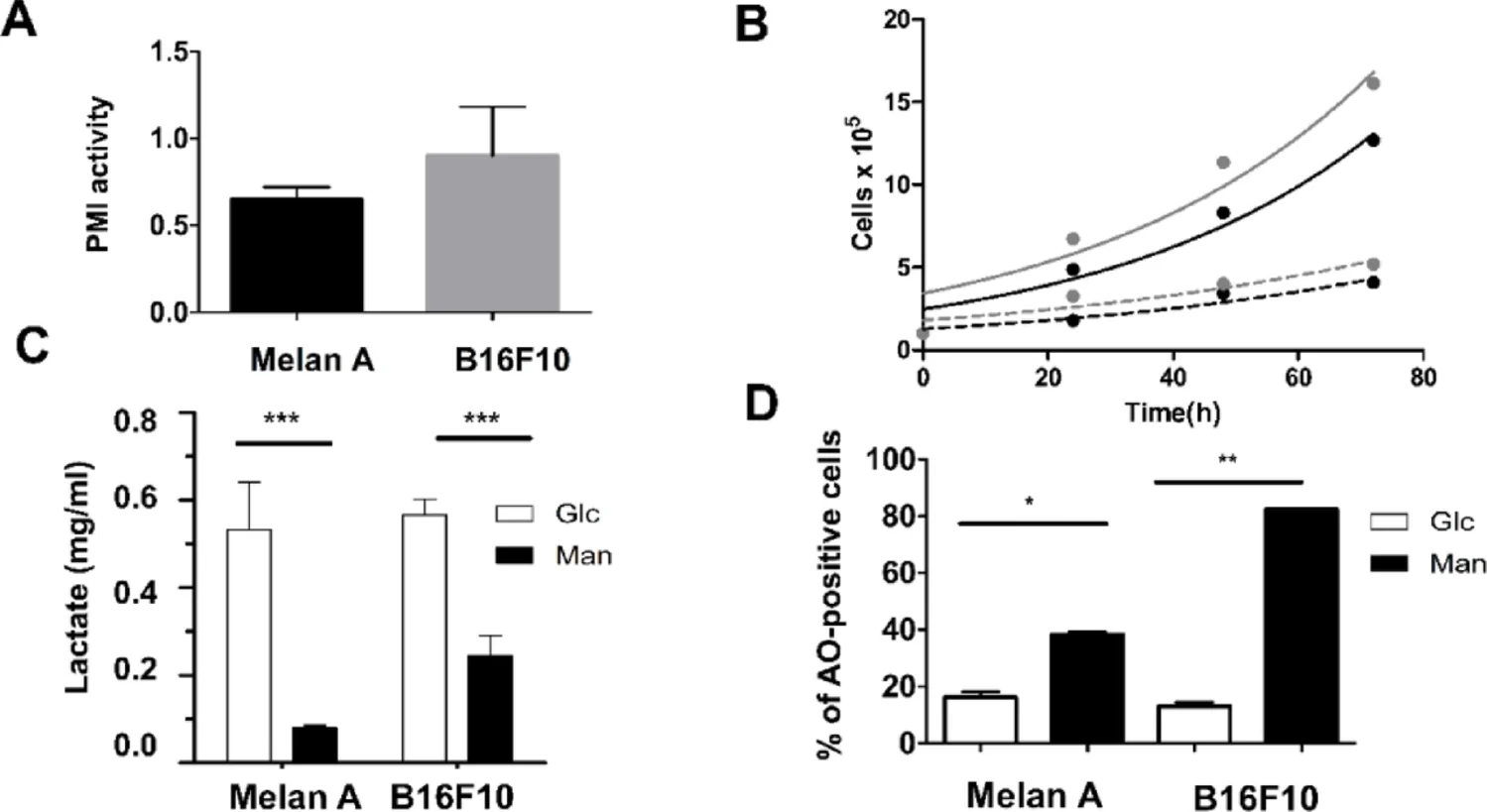
Phenotypic characterization of the effects of mannose on normal melanocytes (Melan-A) and metastatic melanoma cells (B16F10). **A** Phosphomannose isomerase (PMI) enzymatic activity in Melan-A and B16F10 cells. **B** Growth curves of Melan-A (dashed lines) and B16F10 (solid lines) cells cultured in glucose- (gray) or mannose-containing (black) media. **C** Lactate concentration in the culture media of Melan-A and B16F10 cells grown under glucose or mannose conditions. **D** Percentage of acridine orange (AO)-positive cells after culture in glucose- or mannose-containing media, indicating the accumulation of acidic vesicles. Statistical significance is indicated as *P ≤ 0.05, **P ≤ 0.01, and ***P ≤ 0.001 (unpaired two-tailed Student’s t-test)

According to both transciptome and protein expression repositories [12, 13], the baseline expression profile of phosphomannose isomerase gene (MPI) is similar in murine and humans, with soft tissues such as lung, testis and pancreas displaying high expression levels whereas the lowest levels are often observed in skin tissue. Such a pattern of expression was mirrored in the comparable activity of phosphomannose isomerase enzyme in both normal (Melan-A) and metastatic melanocytes (Fig. 1A). In addition, when normal and metastatic (B16F10) cells were grown in culture medium with mannose as the source of hexose, their growth was equally affected (Fig. 1B). Mannose markedly altered cellular metabolism, as evidenced by changes in lactate accumulation in the culture medium. Compared with glucose, mannose significantly affected lactate production (Fig. 1C). Moreover, melanoma cells cultured in mannose exhibited higher lactate levels than normal melanocytes. A similar pattern has been reported in human melanoma cell lines with distinct mutational backgrounds, in which rapidly proliferating A375 cells displayed higher lactate production regardless of the hexose source in the culture medium [8]. Staining with the acidotropic dye acridine orange (AO) revealed an increased abundance of acidic vesicles after 24 h of culture in mannose-containing medium in both cell lines, with metastatic melanoma cells exhibiting the highest proportion of AO-positive cells (Fig. 1D).

This finding is consistent with a cellular stress response elicited by mannose, which can impair glycolytic flux and disrupt energy homeostasis. Under conditions of metabolic stress, the accumulation of acidic vesicles is commonly associated with autophagosome formation and lysosomal activity, promoting the degradation and recycling of intracellular components to sustain cellular energy demands, particularly in rapidly proliferating or nutrient-deprived cells [23, 24]. It should be noted that the complete replacement of glucose by mannose represents a stronger metabolic challenge than would be expected under therapeutic conditions. However, the objective of this experimental design was not to mimic a clinical intervention, but rather to maximize mannose-induced metabolic stress in order to characterize the molecular adaptation programs engaged by normal and metastatic melanocytic cells.

Collectively, these findings suggest that the functional status of phosphomannose isomerase influences the biological responses of normal melanocytes and melanoma cells to mannose exposure.

### Proteome (and phosphoproteome) remodeling under mannose culture conditions

**Fig. 2:**
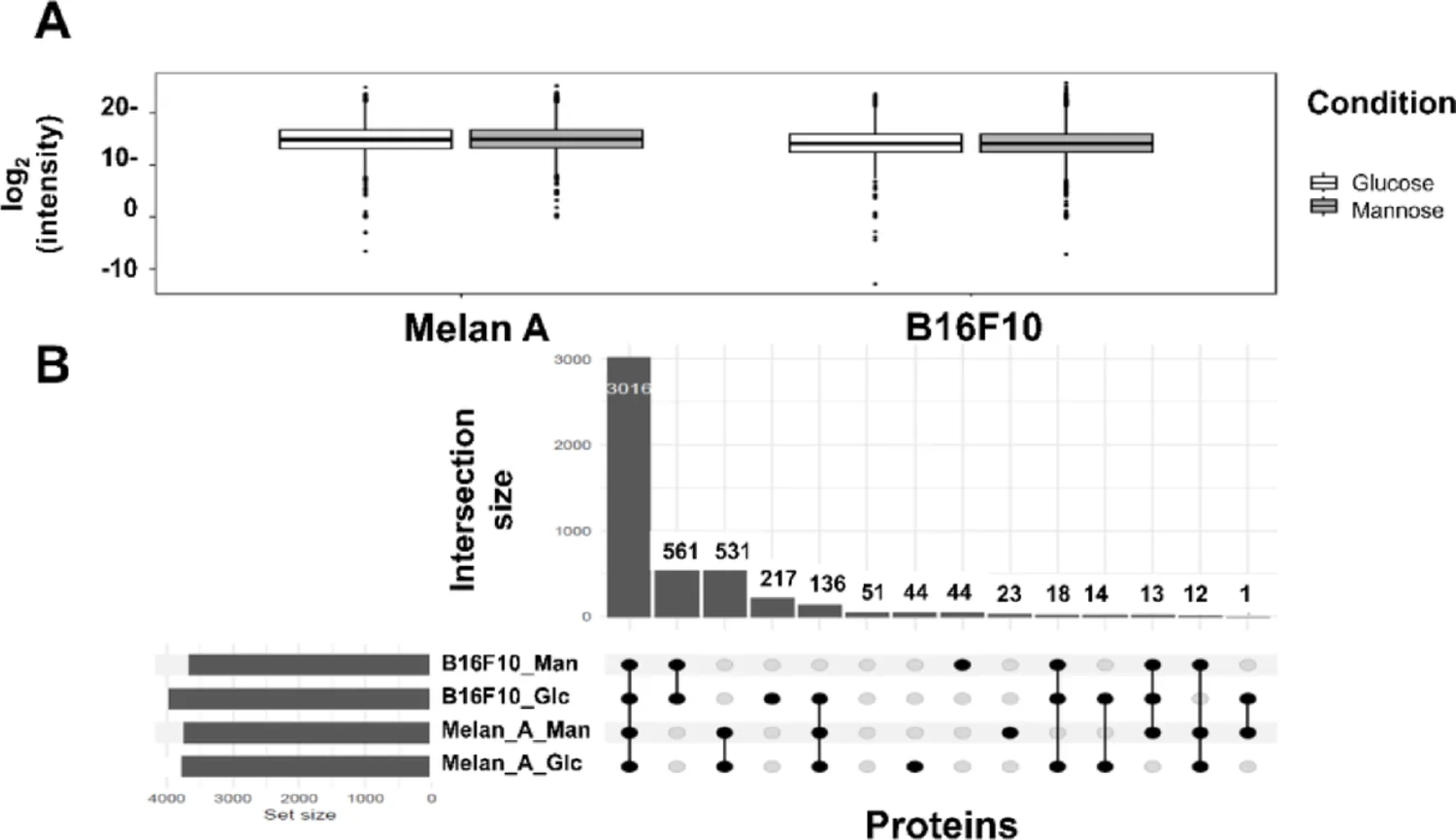
Quantitative proteomic analysis of normal melanocytes (Melan-A) and metastatic melanoma cells (B16F10) cultured in glucose- or mannose-containing media. **A** Distribution of protein abundances (log_2_-transformed protein intensities) across all experimental conditions and cell lines. **B** UpSet plot showing the intersections of identified proteins among the different experimental conditions

Although mannose elicited similar phenotypic effects in normal and transformed melanocytes, we next investigated the molecular features associated with these responses by comparing their proteomic landscapes. To this end, we performed a shotgun proteomics analysis of normal melanocytes and metastatic melanoma cells cultured in media containing either mannose or glucose as the primary hexose source. Protein abundance profiles and the number of identified proteins were comparable among cell lines grown under both hexose conditions (Fig. 2A and B).

**Fig. 3:**
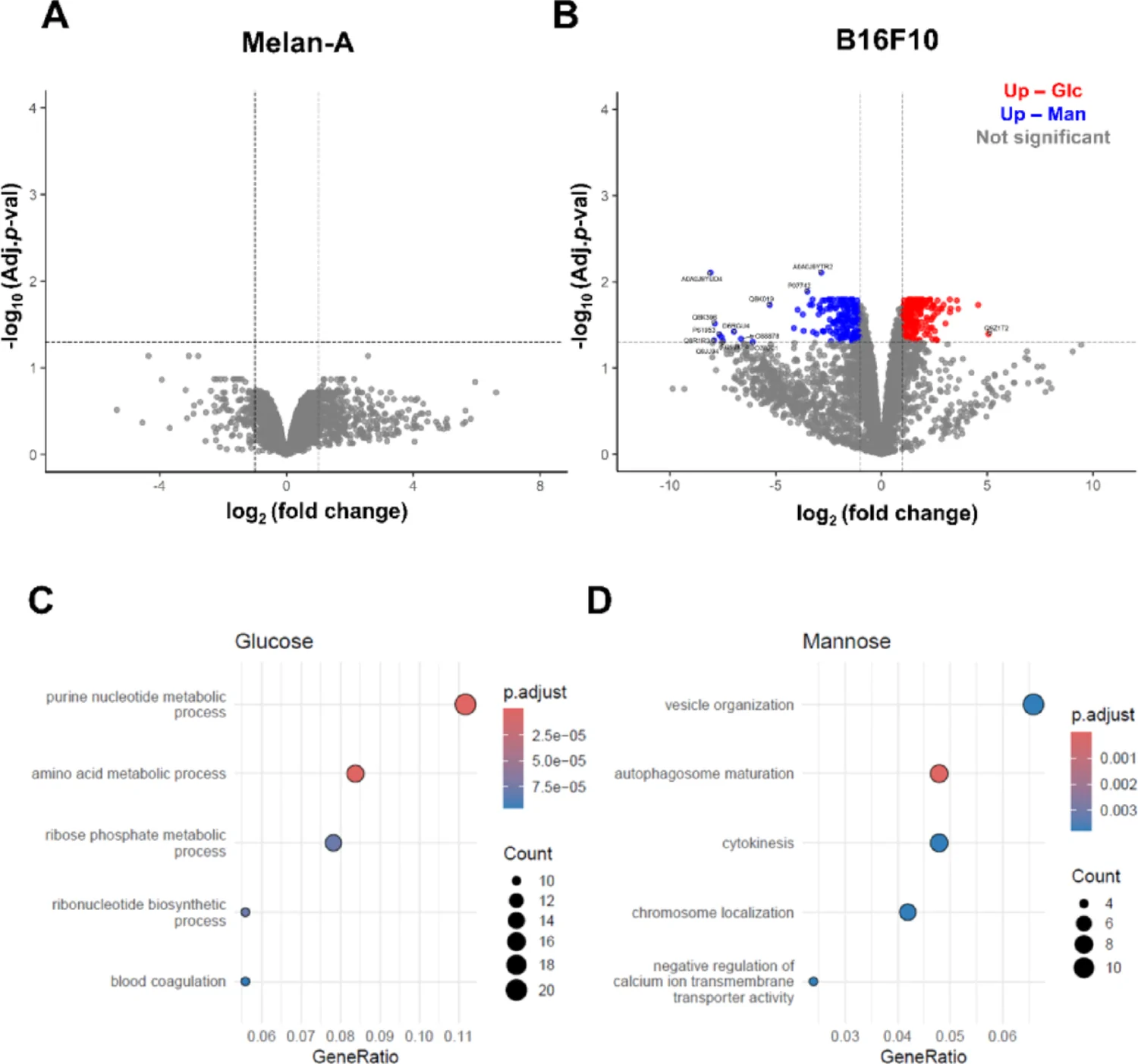
Functional landscape of mannose-induced proteomic remodeling in normal melanocytes (Melan-A) and metastatic melanoma cells (B16F10). Volcano plots showing differential protein abundance between glucose (25 mM) and mannose (25 mM) culture conditions in **A** Melan-A and **B** B16F10 cells. Protein abundance is represented as log_2_ fold change, and statistical significance was determined using linear modeling with empirical Bayes moderation (limma), followed by Benjamini–Hochberg adjustment for multiple testing. Red dots indicate proteins with higher abundance under glucose conditions, whereas blue dots indicate proteins enriched under mannose conditions. UniProt accession numbers are shown for differentially abundant proteins. No proteins reached statistical significance in Melan-A cells after multiple-testing correction. Gene Ontology (Biological Process) enrichment analysis of differentially abundant proteins identified in B16F10 cells cultured under **C** glucose and **D** mannose conditions

In total, more than 3,000 proteins were identified (Supplementary Tables S1 and S2), with good reproducibility among biological replicates, as indicated by the Pearson correlation coefficients (Supplementary Fig. S1). Differential abundance analysis was performed to compare glucose and mannose culture conditions. Although no proteins reached statistical significance in Melan-A cells, several proteins exhibited marked abundance changes (|log_2_ fold change| > 1). While these changes should be interpreted with caution, Gene Ontology enrichment analysis of these proteins is presented in Supplementary Figure S2 to provide additional biological context for the molecular response of normal melanocytes to mannose. Notably, the enriched biological processes differed from those observed in metastatic melanoma cells. In contrast, differential protein abundance analysis of the metastatic cell line B16F10 revealed 373 proteins with significantly altered abundance (Fig. 3A and B; Supplementary tables S3 and S4). Cells cultured in glucose displayed up-regulation of proteins predominantly associated with biosynthetic pathways, including protein synthesis and carbohydrate anabolic processes (Fig. 3C), reflecting an anabolic state that supports cellular growth and homeostasis. In contrast, cells grown in mannose exhibited a proteomic profile consistent with a nutrient-deprived state, characterized by enrichment of proteins involved in autophagosome maturation, vesicle organization, and related adaptive processes (Fig. 3D). These results suggest that, although mannose induced substantial alterations in metabolic circuitry in both cell phenotypes, metastatic cells exhibited greater adaptive plasticity in response to this challenging environment.

**Fig. 4:**
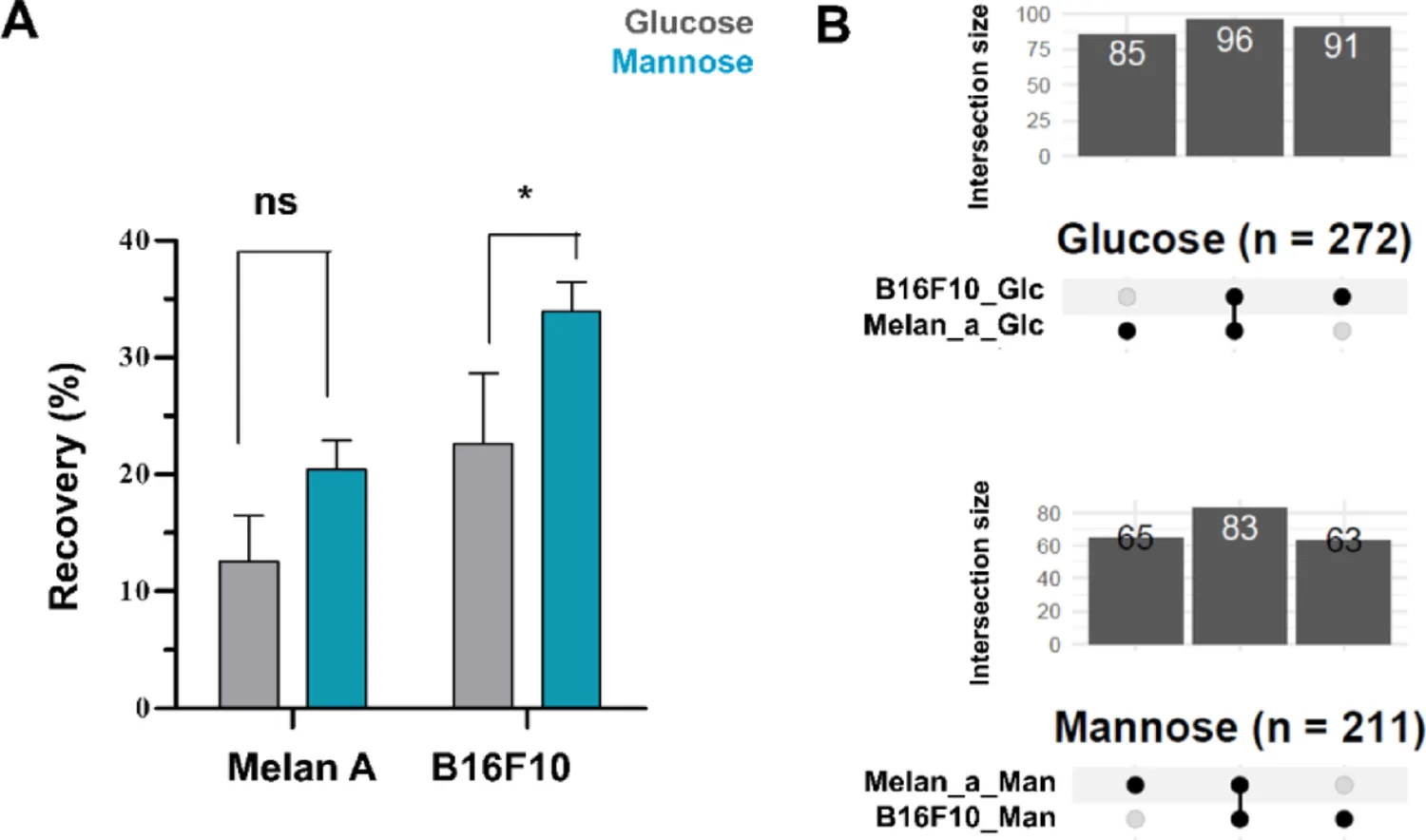
Global phosphoproteomic profiling of normal melanocytes (Melan-A) and metastatic melanoma cells (B16F10) cultured under glucose or mannose conditions. **A** Recovery of phosphoproteins following phosphoprotein enrichment in Melan-A and B16F10 cells cultured in glucose- or mannose-containing media. Mannose treatment increased the overall recovery of phosphoproteins in both cell lines, with a more pronounced effect in metastatic melanoma cells. **B** UpSet plots showing the overlap of phosphoproteins identified in Melan-A and B16F10 cells cultured under glucose (upper panel) or mannose (lower panel) conditions. A total of 272 and 211 phosphoproteins were identified under glucose and mannose conditions, respectively. The plots highlight the phosphoproteins shared between the two cell lines as well as those uniquely identified in each cellular context

Since protein kinases orchestrate a wide range of metabolic and signaling processes, and their activity is extensively remodeled during malignant transformation, we sought to characterize the phosphoproteome of both cell types under the experimental conditions described above. The results of phosphoprotein recovery (following the enrichment procedure) suggested that mannose increased phosphoprotein recovery in both cell lines, with this effect being significantly pronounced in the metastatic B16F10 cell (Fig. 4A). The enrichment procedure led to the identification of a comparable number of phosphoproteins (~ 300 proteins identified at 1% FDR at both the protein and peptide levels; Supplementary tables S5 and S6; Supplementary figure S3) across both experimental conditions and cell lines (Fig. 4B).

Remarkably, in relation to phosphoproteins, both hexoses invoked distinct cellular responses in either cell line and no overlap was observed among the exclusive proteins identified in mannose condition. Although approximately one-third of the identified phosphoproteins were shared between Melan-A and B16F10 cells under each culture condition, a substantial proportion remained cell line-specific, indicating that the identified phosphoproteome comprises a conserved core together with distinct cell context-dependent phosphoproteomic signatures. It should be noted, however, that the phosphoproteomic differences observed between Melan-A and B16F10 cells may reflect not only the biological distinction between normal and metastatic melanocytic cells but also the intrinsic PMA requirement of the Melan-A cell line, which constitutes an inherent characteristic of this experimental model. Nevertheless, these findings indicate that the two cell types engage distinct phosphoproteomic adaptation programs when challenged with the same metabolic perturbation.

**Fig. 5:**
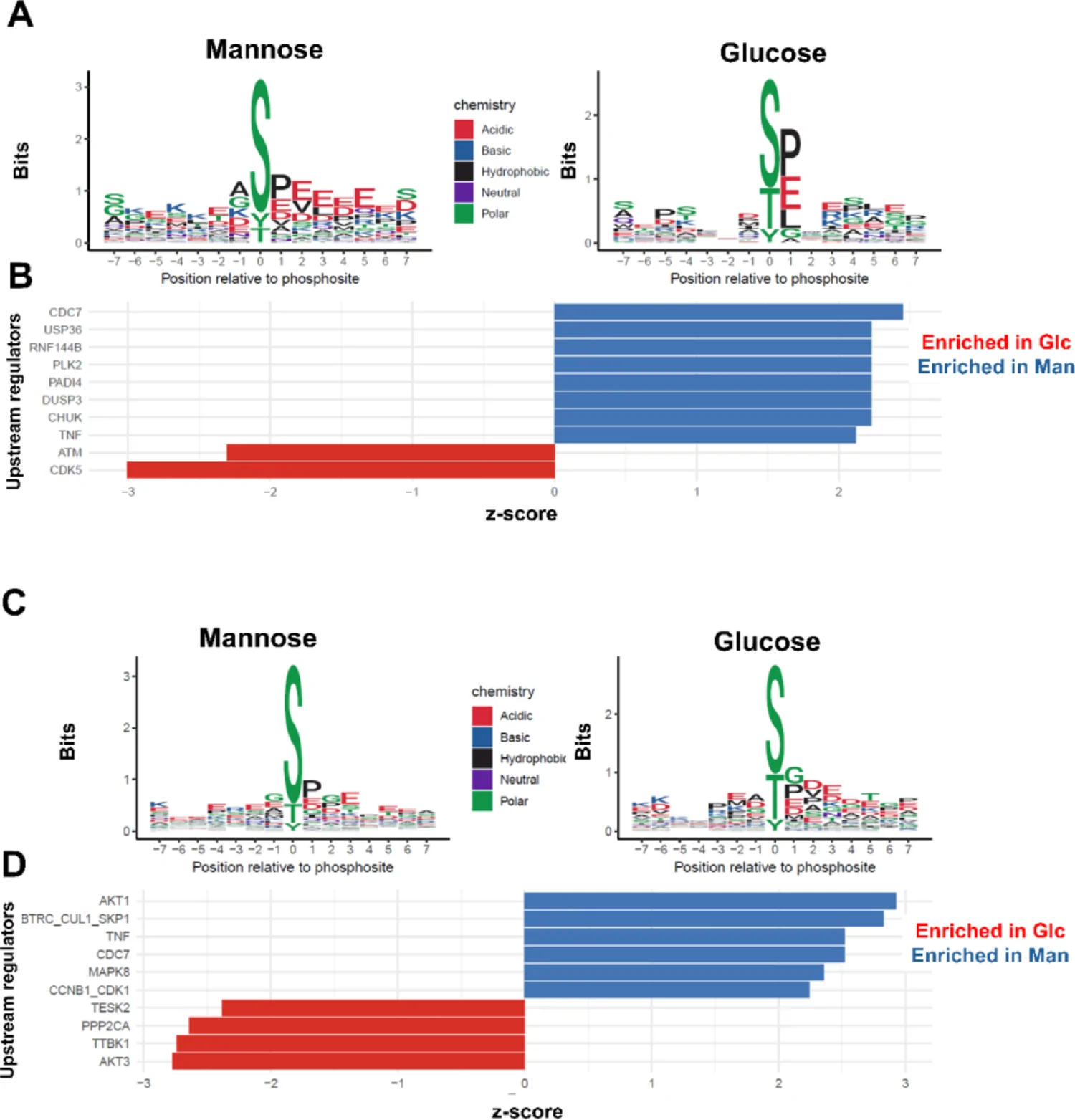
Exploratory substrate enrichment analysis based on phosphoproteomic data. Positional residue enrichment analysis showing the predominant phosphorylation motifs surrounding phosphosites in normal melanocytes (Melan-A) and metastatic melanoma cells (B16F10) (**A** and **C**, respectively) cultured under mannose and glucose conditions. The enriched motifs were used to infer the predominant classes of protein kinases associated with each condition. **B** and **D** Substrate enrichment analysis integrating experimentally identified phosphorylated substrates with curated signaling interactions from OmniPath for Melan-A and B16F10 cells, respectively. Upstream regulators are ranked according to their standardized *z*-scores, with positive values indicating preferential enrichment under mannose conditions (blue bars) and negative values indicating preferential enrichment under glucose conditions (red bars)

Positional residue enrichment analysis was performed to infer kinase classes based on canonical phosphorylation motifs. In Melan-A cells cultured under glucose conditions, the enrichment of proline and glutamic acid residues surrounding the phosphosite suggested the activity of proline-directed kinases, such as mitogen-activated protein kinases (MAPKs) and cyclin-dependent kinases (CDKs). In contrast, under mannose treatment, the recurrent enrichment of proline and acidic residues suggested not only the activity of proline-directed kinases but also the involvement of acidophilic kinases (Fig. 5A). Furthermore, substrate enrichment analysis integrating experimentally identified phosphorylated substrates with curated signaling interactions predicted distinct upstream regulatory profiles between the two experimental conditions.

Under glucose conditions, substrate enrichment analysis identified phosphosubstrate signatures associated with ATM serine/threonine kinase (ATM) and cyclin-dependent kinase 5 (CDK5) (Fig. 5B). Conversely, under mannose conditions, phosphosubstrate signatures were associated with cell division cycle 7-related protein kinase (CDC7), conserved helix–loop–helix ubiquitous kinase (CHUK/IKKα), serine/threonine-protein kinase PLK2, and additional upstream regulators, consistent with extensive phosphoregulatory remodeling in response to mannose exposure. In metastatic melanoma cells, positional residue enrichment analysis revealed phosphorylation signatures consistent with proline-directed and acidophilic phosphorylation motifs under mannose treatment, mirroring the pattern observed in mannose-treated normal melanocytes. In contrast, metastatic cells cultured under glucose exhibited a pronounced enrichment of acidic residues surrounding the phosphosites, consistent with a predominance of acidophilic phosphorylation motifs under this condition (Fig. 5C). Substrate enrichment analysis also identified distinct phosphosubstrate signatures associated with the two culture conditions. Among the inferred regulator-associated phosphosubstrate signatures, CDC7 and MAPK8 were preferentially associated with mannose conditions, whereas AKT3 and TTBK1 were preferentially associated with glucose conditions (Fig. 5D).

**Fig. 6:**
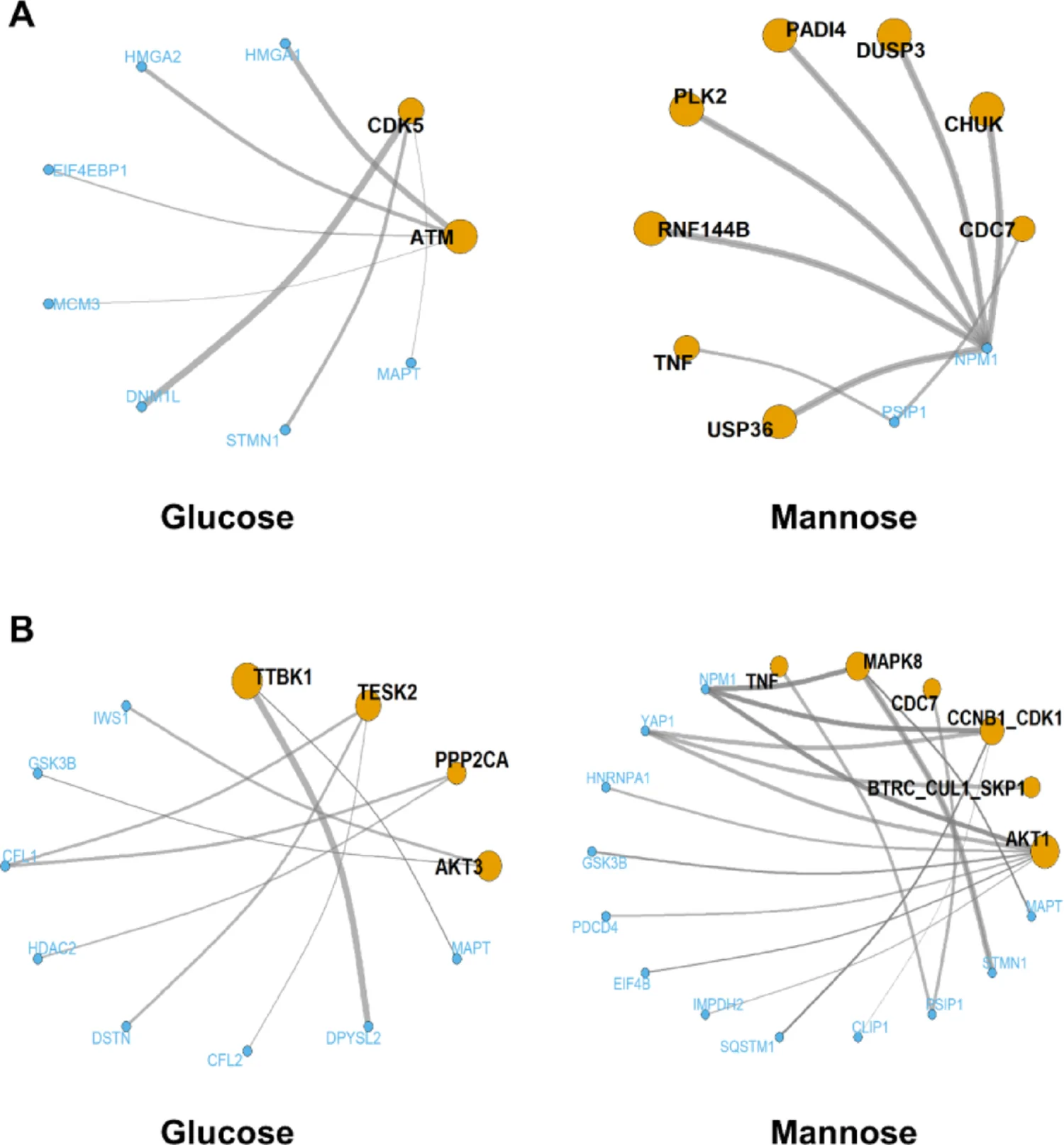
Regulator–substrate interaction networks inferred from phosphoproteomic data. **A** Normal melanocytes (Melan-A) and **B** metastatic melanoma cells (B16F10). Networks were generated using the top 10 upstream regulators identified by substrate enrichment analysis together with their corresponding experimentally identified phosphorylated substrates. For each cell type, upstream regulators were grouped according to their associated phosphosubstrate signatures under glucose or mannose culture conditions, as determined by their standardized *z*-scores. Orange nodes represent inferred upstream regulators, whereas blue nodes represent experimentally identified phosphorylated substrates. Edges denote curated regulator–substrate interactions retrieved from OmniPath, and their width is proportional to the absolute log_2_ fold change of the corresponding phosphorylated substrate. Node size reflects the degree of connectivity (number of interactions), highlighting highly connected upstream regulators and phosphorylated substrates within each regulatory network

Network analysis of the inferred regulator–substrate relationships further illustrated the distinct phosphoregulatory programs associated with each culture condition (Fig. 6). Networks were constructed using the top-ranked upstream regulators identified by substrate enrichment analysis and their corresponding experimentally identified phosphorylated substrates. Upstream regulators associated with glucose or mannose were displayed separately, while edge thickness was scaled according to the absolute log_2_ fold change of each phosphorylated substrate. In normal melanocytes, substrate enrichment analysis identified phosphosubstrate signatures associated with CDK5 and ATM under glucose conditions, whereas under mannose, phosphosubstrate signatures were associated with CHUK, CDC7, DUSP3, PADI4, PLK2, RNF144B, TNF, and USP36 (Fig. 6A). These inferred regulator–substrate relationships connected distinct upstream regulators to subsets of experimentally identified phosphorylated substrates, indicating extensive remodeling of phosphoregulatory networks following mannose treatment.

A similar condition-dependent reorganization was observed in metastatic melanoma cells (Fig. 6B). Under glucose conditions, phosphosubstrate signatures were associated with AKT3, PPP2CA, TESK2, and TTBK1, whereas under mannose, they were associated with AKT1, CCNB1/CDK1, CDC7, MAPK8, TNF, and the β-TrCP/CUL1/SKP1 complex (BTRC_CUL1_SKP1). Several phosphorylated substrates were shared among multiple upstream regulators, highlighting the interconnected nature of the regulatory network and suggesting coordinated control of phosphorylation events under each metabolic condition. Collectively, these networks illustrate that mannose induces extensive remodeling of inferred regulator–substrate relationships in both normal melanocytes and metastatic melanoma cells, although the specific upstream regulators and experimentally identified phosphorylated substrates involved differ markedly between the two cellular contexts.

### Functional consequences of mannose-induced remodeling in metastatic melanoma cells

In melanoma cells, the functional status of PMI enzyme has been shown to shape the proteomic and degradomic responses to mannose treatment, highlighting the biological importance of mannose metabolism in this cellular context [8]. The low abundance of phosphomannose isomerase has important functional consequences for melanocyte biology. Because PMI catalyzes the conversion of mannose-6-phosphate (M6P) into fructose-6-phosphate (F6P), limited PMI availability restricts this metabolic step, leading to the intracellular accumulation of M6P. Since mannose can compete with glucose for hexokinase and suppresses glycolysis [25], accumulated M6P may contribute to hexokinase inhibition, thereby impairing glycolytic flux [5, 26]. The present study complements our previous findings by providing the first comparative molecular characterization of normal and malignant melanocytic cells exposed to mannose. Nevertheless, the translational potential of mannose-based metabolic interventions will require further investigation in clinically relevant models, particularly considering the functional status of phosphomannose isomerase (PMI), which may represent an important determinant of therapeutic responsiveness.

Phenotypic analyses following mannose treatment—including cell growth, lactate production, and the accumulation of acidic vesicles—revealed remarkably similar responses in normal melanocytes and melanoma cells. However, subsequent proteomic and phosphoproteomic analyses uncovered distinct molecular adaptations, indicating that despite their shared phenotype, normal and transformed cells engage fundamentally different mechanisms to cope with mannose-induced metabolic stress. Consistent with this observation, the phosphoproteomic response to mannose was highly cell context-dependent, promoting predominantly homeostatic signaling in normal melanocytes while eliciting extensive signaling rewiring in metastatic melanoma cells. Our phosphoproteomic data suggest that glucose is associated with a phosphoregulatory landscape characteristic of metastatic melanoma. In particular, substrate enrichment analysis revealed distinct phosphosubstrate signatures associated with AKT family members, with substrates linked to AKT3 being more prominently represented under glucose conditions, whereas substrates associated with AKT1 predominated under mannose conditions. Although these computational inferences require direct experimental validation, they suggest that distinct AKT-centered regulatory modules may contribute to the adaptive response elicited by mannose in melanoma. Rather than simply suppressing cellular signaling, mannose promoted extensive phosphoproteomic remodeling, with substrate enrichment analysis identifying phosphosubstrate signatures associated with regulators involved in stress responses (MAPK8/JNK), adaptive signaling (AKT1), cell-cycle regulation (CDC7 and CCNB1/CDK1), and ubiquitin-mediated protein turnover (BTRC–CUL1–SKP1). Collectively, these findings support the hypothesis that metastatic melanoma cells undergo coordinated phosphoregulatory remodeling to adapt to mannose-induced metabolic stress.

Mannose therefore appears to challenge metastatic melanoma cells by promoting a coordinated adaptive response to mannose-induced metabolic stress, rather than suppressing their signaling network. The coexistence of a conserved phosphoproteomic core and extensive cell-specific phosphorylation patterns reinforces the notion that similar phenotypic responses to mannose arise from distinct molecular adaptation programs shaped by cellular context. Taken together, our data support a model in which mannose-induced metabolic stress is accommodated through coordinated remodeling of both the proteome and phosphoproteome, consistent with an adaptive response in metastatic melanoma cells characterized by extensive changes in the inferred phosphoregulatory landscape.

In summary, our results establish that the cellular response to mannose is highly context-dependent and reveal that adaptation to metabolic stress involves coordinated proteomic and phosphoproteomic remodeling rather than a simple suppression of signaling pathways. Although mannose elicited convergent phenotypic responses in normal melanocytes and metastatic melanoma cells, these responses were underpinned by fundamentally distinct molecular adaptation programs, highlighting cell context as a key determinant of the response to mannose. Overall, our findings provide new molecular insights into the cellular response to mannose in melanoma and identify coordinated molecular remodeling as a hallmark of the adaptive response to mannose, providing a molecular framework for future studies exploring metabolic vulnerabilities and combinatorial therapeutic strategies in melanoma.

## Supplementary Information

Below is the link to the electronic supplementary material.

Supplementary Material 1

Supplementary figures

## Data Availability

The mass spectrometry proteomics data have been deposited to the Mass Spectrometry Interactive Virtual Environment (MassIVE) https://massive.ucsd.edu/ProteoSAFe/static/massive.jsp) with the dataset identifier MSV000102497.
